# Impact of hip fracture on survival, disability, pain, and health-related quality of life in Zimbabwe: a prospective cohort study

**DOI:** 10.1016/j.lanhl.2025.100766

**Published:** 2025-10-11

**Authors:** Mohamad I Nasser, Anya Burton, Hannah Wilson, Tadios Manyanga, Tafadzwa Madanhire, Prudance Mushayavanhu, Munyaradzi Ndekwere, Joseph Chipanga, Samuel Hawley, Simon Matthew Graham, James Masters, Kate A Ward, Matthew L Costa, Rashida A Ferrand, Celia L Gregson

**Affiliations:** Musculoskeletal Research Unit, Bristol Medical School, University of Bristol, Southmead Hospital, Bristol, UK; The Health Research Unit Zimbabwe at the Biomedical Research and Training Institute, Harare, Zimbabwe; Musculoskeletal Research Unit, Bristol Medical School, University of Bristol, Southmead Hospital, Bristol, UK; The Health Research Unit Zimbabwe at the Biomedical Research and Training Institute, Harare, Zimbabwe; Department of Surgery, Sally Mugabe Central Hospital, Harare, Zimbabwe; Department of Surgery, Midlands State University, Gweru, Zimbabwe; Department of Surgery, Midlands State University, Gweru, Zimbabwe; The Health Research Unit Zimbabwe at the Biomedical Research and Training Institute, Harare, Zimbabwe; Musculoskeletal Research Unit, Bristol Medical School, University of Bristol, Southmead Hospital, Bristol, UK; Oxford Trauma and Emergency Care, Nuffield Department of Orthopaedics, Rheumatology and Musculoskeletal Science, University of Oxford, Oxford, UK; MRC Lifecourse Epidemiology Centre, Human Development and Health, University of Southampton, Southampton, UK; MRC Unit, The Gambia at London School of Hygiene & Tropical Medicine, Banjul, The Gambia; Oxford Trauma and Emergency Care, Nuffield Department of Orthopaedics, Rheumatology and Musculoskeletal Science, University of Oxford, Oxford, UK; The Health Research Unit Zimbabwe at the Biomedical Research and Training Institute, Harare, Zimbabwe; Clinical Research Department, London School of Hygiene & Tropical Medicine, London, UK; Musculoskeletal Research Unit, Bristol Medical School, University of Bristol, Southmead Hospital, Bristol, UK; The Health Research Unit Zimbabwe at the Biomedical Research and Training Institute, Harare, Zimbabwe

## Abstract

**Background:**

The population in Africa is ageing, and fragility fractures increasing. We assessed 1-year health outcomes following hip fracture in older adults in Zimbabwe.

**Methods:**

In this prospective cohort study, a cohort of adults aged 40 years or older with hip fracture, presenting to hospitals in Harare (two public and five private hospitals) between Oct 15, 2021, and Oct 14, 2022, were followed up for 12 months. The primary outcome was survival, analysed with Kaplan–Meier curves at different timepoints (30 days, 120 days, 6–8 months, and 12 months after case identification), overall and stratified by age (<70 years *vs* ≥70 years), delay to presentation (no delay [≤2 weeks] *vs* delay [>2 weeks]), and facility type and operative management. We also quantified health-related quality of life (HRQoL), measured with 5-level EQ-5D (EQ-5D-5L), hip pain, self-reported from 0 (none) to 5 (all the time) and measured as interference with walking and sleep (1 [no interference] to 10 [complete interference]), as per the Brief Pain Inventory, and disability, measured with the WHO Disability Assessment Schedule version 2.0 (WHODAS).

**Findings:**

Of 196 patients with hip fracture (96 [49%] female, 100 [51%] male; median age 74 years [IQR 62⋅5–83]), 162 (83%) had had a fragility fracture (low-energy trauma). In total, 173 (88%) were managed in a public hospital, of whom 96 (55%) received operative hip fixation. In contrast, all of the 23 (12%) managed in private facilities had an operation. After 12 months, 55 (29%) had died (49 [42%] of 117 patients aged ≥70 years, and six [9%] of 70 patients aged <70 years). In public hospitals, 31 (42%) of 73 non-operated patients died, compared with 18 (19%) of 93 patients who were operated on. Overall, survival declined to 88% (95% CI 82–92) by 30 days and to 71% (64–77) by 12 months. The probability of survival was lower in patients aged 70 years or older than in those younger than 70 years (mortality hazard ratio for ≥70 years 6⋅10, 95% CI 2⋅61−14⋅22). The mean HRQoL utility score decreased from 0⋅81 (95% CI 0⋅80–0⋅83) pre-fracture to 0⋅29 (0⋅25–0⋅34) at 30 days post fracture. Minimal recovery was seen after 120 days (0⋅34, 0⋅29–0⋅39). By 12 months, 97 (97%) of 100 patients alive and able to provide data still reported pain from their hip injury. Post-fracture disability was almost universal, with only two (2%) of 100 patients being disability-free (WHODAS=0) by 12 months.

**Interpretation:**

Following hip fracture, survival and quality of life decreased substantially in the study population. These findings reveal the need for the implementation of guidelines to standardise care and improve operative capacity to manage the predicted rise in fractures in this region.

**Funding:**

Wellcome Trust.

## Introduction

Populations are ageing across Africa, with the number of older adults (aged ≥ 60 years) in the sub-Saharan region expected to have increased from 46 million in 2015 to 161 million by 2050.^1^ This demographic shift, driven by improving living conditions,^2^ health-care access, and thus longevity,^3^ is increasing the prevalence of age-related health conditions, including musculoskeletal disease such as osteoporosis.^4^ Osteoporosis remains silent until a fragility fracture occurs, the most devastating of which is a hip fracture.^2^ Fragility fractures are associated with high morbidity, mortality, and economic cost.^5^ In high-income countries, after a hip fracture, reports of 1-year mortality range from 22% to 28⋅2%,^6,7^ health-related quality of life (HRQoL) is profoundly and permanently reduced,^8^ and disability is common, leading to loss of independence.^9^ These adverse health outcomes have driven clinical practice standards towards prompt surgery (within 36 h of admission) for almost all cases, unrestricted weightbearing mobilisation (within a day of surgery), and complex multidisciplinary care (eg, comprehensive geriatric assessment by a multidisciplinary team within 3 days of admission).^10^

Few studies have examined health outcomes after hip fracture in African countries. The limited research available suggests that outcomes, such as mortality rates, might be worse than in high-income regions. In a Ghanaian study of hip fractures, 15 (20%) of 76 patients had died within a year,^11^ whereas in South Africa, a larger study in public hospitals identified a 1-year mortality of 33⋅5% (67 of 200 patients died within a year).^12^ Long delays were documented from admission to surgery (median 19⋅0 days [IQR 12⋅3–25⋅0]).^12^ In this context, multiple factors will influence fracture care provision and outcomes: socio-economic deprivation, malnutrition, a high HIV prevalence and associated use of antiretroviral therapy (some of which have adverse effects on bone health), and challenged health-care infrastructure.^2,13–15^ An example of such a context is Zimbabwe, a lower-middle-income country with the highest hyperinflation globally, and where 38⋅4% of the population live below the extreme poverty line.^16,17^

To provide actionable insights towards improved healthcare policy and practice, and patient outcomes, we aimed to determine health-related outcomes in the year following hip fracture, also assessing survival, disability, and pain in a cohort of older adults who sustained a hip fracture in Zimbabwe. We further aimed to understand operative management practices and how receipt of surgical treatment was associated with health-related outcomes after hip fracture.

## Methods

### Study design and population

This prospective cohort study was conducted in Harare, the capital city of Zimbabwe, where an estimated 362 524 adults aged 40 years and older reside in a largely peri-urban setting.^18^ The study forms part of an international research programme on fracture care (Fractures-E3).^19^ This report has been prepared in accordance with STROBE guidelines.^20^

Eight hospitals provide all hip fracture care in the city of Harare and neighbouring rural provinces; both public hospitals and five of the six private hospitals agreed to onsite data collection. One small private hospital that would rarely have seen hip fractures, if at all, declined. Over 1 year (from Oct 15, 2021, to Oct 14, 2022), we identified all patients aged 40 years or older with an incident hip fracture, presenting to one of these seven hospitals. We were granted ethics approval to collect a basic dataset of key variables from medical records on all people experiencing a hip fracture, before approaching all for informed consent, to calculate the incidence of hip fracture (age, sex, region of residence, presentation date, time since injury, hip fracture classification, and trauma mechanism).^21^ If a patient died before being approached for consent, this was recorded. All patients were then invited to participate in this cohort study. Consenting patients were followed up for 12 months, with data collected at up to five timepoints where possible: during admission (of which 70% were within a week of presentation), at 30 days, at 120 days, between 6 and 8 months, and lastly 12 months from date of case identification when the basic dataset was completed. Those who died before they could provide consent were included in analyses where only data from the basic dataset was needed (survival analyses and odds of operative fixation), to prevent bias. Following hospital discharge, follow-up visits were conducted in patients’ place of residence, except for the 6–8-month visit, which was mainly done via telephone. For those in distant rural locations, telephone follow-up at any timepoint was possible. Through caregiver networks, ascertainment of vital status at 12 months (alive, dead, or censored [consent withdrawn or lost to follow-up]) was prioritised.

All patients provided written or thumb print informed consent to participate in the cohort study. For those who lacked capacity to consent, proxy assent was sought from a family member or caregiver, so as not to exclude patients with delirium or dementia. Ethics and governance approvals were obtained from the Medical Research Council of Zimbabwe, the Biomedical Research and Training Institute, the Sally Mugabe Central Hospital, the University of Zimbabwe College of Health Sciences and the Parirenyatwa group of hospitals, Harare City Health, and the Research Council of Zimbabwe (appendix 2 p 2).

### Hip fracture detection and validation

Hip fractures were confirmed by radiographs. All radiographs were reviewed by two consultant orthopaedic surgeons. Each fracture was classified as intracapsular (ICD-10 code S72.0), intertrochanteric (S72.1), or sub-trochanteric (S72.2). Where no radiograph was available (an anticipated event due to x-ray equipment faults, interrupted electricity supply, unaffordability of radiographs, or missing x-ray films), the mode of injury, patient symptoms, and examination findings were used to verify a hip fracture by an orthopaedic surgeon. The mode of injury and symptoms included (1) a fall in which the patient landed on their buttocks or side, after which (2) they quickly experienced severe pain in the groin or hip with or without radiation to the knee, combined with (3) a shortened and externally rotated leg on examination. The presence of all three conditions were used to make a clinical diagnosis of hip fracture. If a radiograph had been taken, but was not made available to the research team, the hip fracture classification was taken from the orthopaedic diagnosis documented in the hospital medical records.

### Participant characteristics and hospital management

Medical record reviews and researcher-administered questionnaires collected data on age, sex, residence, education, marital status, household income, tobacco use, alcohol consumption, HIV status (if known and willing to disclose), mechanism of injury (low-energy trauma [eg, fall from a standing height or less] *vs* high-energy trauma [eg, road traffic accident]), delay in presentation (>2 weeks since injury), and date of death. Hospital data collected included facility type (public *vs* private facility), length of hospital stay, whether the hip fracture was managed by surgical operation, and if it was, the American Society of Anesthesiologists grade at operation. Mid-upper arm circumference (MUAC) was measured as an indicator of malnutrition (defined as ≤ 23 cm).^22,23^

Data were entered directly into pre-programmed Research Electronic Data Capture (REDCap) questionnaires with inbuilt data validation, working offline on Samsung Galaxy tablets. REDCap is a secure, web-based software platform designed to support data capture for research studies.^24,25^

### Health-related outcomes

Using patient-reported questionnaires, HRQoL was quantified by the 5-level EQ-5D (EQ-5D-5L), which assesses five health dimensions: mobility, self-care, usual activities, pain or discomfort, and anxiety or depression.^26^ During admission, all patients were asked to recall their pre-injury HRQoL,^27^ after which HRQoL was assessed at each follow-up timepoint. Hip injury pain was assessed (from grade 0 [none] to 5 [all the time]), and, for those who had hip injury pain, participants graded pain interference with sleep and walking ability (1 [no interference] to 10 [complete interference]), as per the Brief Pain Inventory.^28^ Disability was quantified by the WHO Disability Assessment Schedule version 2.0 (WHODAS).^29^ Both pain and disability were assessed at every follow-up timepoint.

### Statistical analysis

Categorial variables are presented as absolute numbers and percentages and continuous variables as median (IQR). We used *χ*^2^ testing to compare proportions and two-sided Wilcoxon and Kruskal-Wallis testing to compare median values across two and three groups, respectively.

Follow-up was defined as the time from date of case identification to 365 days, or until loss to follow-up (date of last contact) or death, whichever occurred first.

For our primary outcome, survival, we used Kaplan–Meier curves to calculate survival probabilities at different timepoints (30-day, 120-day, 6–8-month and 12-month timepoints) overall and stratified by age (<70 years *vs* ≥70 years), delay to presentation (no delay [≤ 2 weeks] *vs* delay [>2 weeks]), and facility type and operative management. Numbers at risk and numbers censored at each timepoint were calculated. To minimise survival bias, those who died before consent were included in survival analyses. To test for differences in Kaplan–Meier curves by groups, we used log-rank testing and calculated hazard ratios (HRs) to compare mortality rates. We used Cox proportional hazards models to estimate crude and age-adjusted mortality HRs for demographic variables, and lifestyle, clinical, fracture, and hospital characteristics. Proportionality assumptions were checked using Schoenfeld residuals tests and inspection of log-log plots.

Our secondary outcomes were operative fixation (yes or no), and post-fracture HRQoL, pain, and disability recovery over 12 months. Associations between demographic, health, lifestyle, fracture, and hospital characteristics and whether a patient had an operative fixation of their hip fracture using univariate logistic regression. All patients were included in these analyses. For HRQoL, the R-package eq5d calculated a single utility score,^30^ by mapping participants’ self-rated health in terms of mobility, self-care, usual activities, pain or discomfort, and anxiety or depression to the Zimbabwe crosswalk value set.^31^ Compared against prefracture HRQoL, we calculated the mean difference (with 95% CIs) in EQ-5D-5L utility score (death imputed as 0)^32^ at each follow-up timepoint. The analyses were stratified by age (<70 years *vs* ≥ 70 years), delay to presentation (≤ 2 weeks *vs* >2 weeks), and facility type and operative management. In sensitivity analyses, analyses were repeated excluding those who died by the end of follow-up. For those alive and with data available at 30 days, 120 days, 6–8 months, or 12 months, we calculated median (IQR) values for the pain outcomes. Similarly, we transformed the WHODAS sum (scored 0–48) to a score of 0–100 (WHODAS×100/48) for ease of interpretation and calculated median and IQR scores for the four follow-up timepoints. Patients who died before consent or withdrew consent for follow-up were excluded from these analyses (n=10).

Complete case analyses were conducted and missing values displayed in table footnotes. Analyses were conducted using Stata (version 18.0), or R statistical software and R-studio (versions 4.3.3).^33^

### Role of the funding source

The funders had no role in study design, data collection, data analysis, data interpretation, or writing of the report.

## Results

Over 1 year, 232 patients with a hip fracture were identified. 36 patients could not be included in the analyses because either the patient (n=13) or their proxy (n=18) declined to participate, the patient could not be located (n=4), or for other reasons (n=1). Of the remaining 196, six died before being able to consent; as basic characteristics and date of death had been established, they were included in survival and operative fixation analyses with ethics approval. Subsequently, 190 (82%) of 232 patients consented to participate and completed the baseline questionnaire, and their admission data were collected. Four further patients with-drew consent for follow-up (appendix 2 p 3) and, along with the six who died before consent, were excluded from HRQoL, pain, and disability longitudinal analyses. Follow-up appointments were missed for 28 (17%) of 165 patients at 30 days and for 37 (27%) of 137 patients at 12 months. Patients were less likely to consent to inclusion in the study at private facilities than at public facilities (21 [48%] of 44 private patients and 15 [8%] of 188 public patients declined consent; appendix 2 p 9).

The median age of the study population was 74 years (IQR 62⋅5–83), 96 (49%) of 196 were female, and 100 (51%) were male (table 1). Median age was 76 years (IQR 70–84) in women and 68 years (52–83) in men. Just over half of patients were resident in Harare, and most patients were living in their own home before admission. The majority had a household income of US$100/month or less, and 21% (40 of 190) had a MUAC of 23 cm or lower, indicative of malnutrition. Overall, 24 (13%) of 190 patients self-reported an HIV diagnosis, 47 (25%) either did not know or did not say, and the rest (119 [63%]) reported having tested HIV-negative.

Most fractures had occurred following a low-energy injury (table 1); 174 (89%) of 196 fractures were radio-graphically confirmed, with intracapsular and intertrochanteric fractures being the most common. Delays of more than 2 weeks in presentation were observed for 72 (37%) of 196 patients. Those who were delayed in presentation were younger, less likely to live in Harare, more likely to present to a public facility, and more likely to have a low income than those who did not present with delay (appendix 2 p 10).

Those who were older (age ≥ 70 years), had lower educational attainment, had lower household income, were more likely to be malnourished and to be widowed, and were less likely to smoke, drink alcohol, and self-report HIV than those who were younger than 70 years (table 1). All but five of those aged 70 years or older had had a low-energy injury consistent with a fragility fracture of the hip, whereas the proportion of patients with high-energy injuries was greater (39%) in those who were younger than 70 years.

Most patients (n=173, 88%) presented to a public hospital (table 1). Overall, only 119 (61%) had an operation for their hip fracture. All 23 patients presenting to a private facility had an operation, compared with 96 (55%) of 173 patients presenting to a public hospital (table 2).

The odds of having an operation were higher for patients with a household income higher than $100 per month than for those with lower incomes (odds ratio 3⋅06, 95% CI 1⋅54–6⋅05) and lower for patients with malnutrition (MUAC ≤ 23 cm; 0⋅31, 0⋅15–0⋅65) and for those with low educational attainment (0⋅41, 0⋅21–0⋅82; table 2). Notably, no patients at private hospitals had a MUAC lower than 23 cm or self-reported living with HIV (table 1).

The median wait in hospital for an operation was 4 days (IQR 2–7⋅5) in private facilities and 20 days (IQR 10–30) in public hospitals (table 1). Whereas patients at private facilities stayed a median of 10⋅5 days (8–13) in hospital, those at public facilities stayed much longer, whether operated on or not (median 24 days). Of the patients who had an operation, those in private facilities stayed a median of 5⋅5 days (3⋅5–8) from operation until discharge, whereas those in public hospitals stayed a median of 3 days (2–5).

Vital status at the end of follow-up (12 months) was missing in nine of the 196 patients (four withdrew consent during follow-up, five could not be contacted). For the 192 who did not withdraw consent, some differences were seen in baseline characteristics between those with known and those with unknown vital status, but due to the low number of participants with unknown vital status, these are difficult to interpret (appendix 2 p 11).

27 (14%) of 187 patients had died by 30 days, and 55 (29%) had died by 365 days (appendix 2 p 12). After 12 months, the proportion of patients who died was higher in patients aged 70 years or older (49 [42%] of 117 patients) than in those younger than 70 years (six [9%] of 70 patients). Survival probability was similar in men and women (appendix 2 p 4). Age was associated with mortality (HR 1⋅06 [95% CI 1⋅03–1⋅08] per year of age; appendix 2 p 5). The proportion of deaths was also higher in those managed non-operatively in a public facility (42%; 31 of 73) than in those managed operatively in a public facility (19%; 18 of 93) or in a private facility (29%; six of 21; appendix 2 p 12). Even after age adjustment, in public facilities the risk of death was nearly three times higher for non-operated patients than those operated on (HR 2⋅87, 95% CI 1⋅61–5⋅12; appendix 2 p 5).

**Figure 1:**
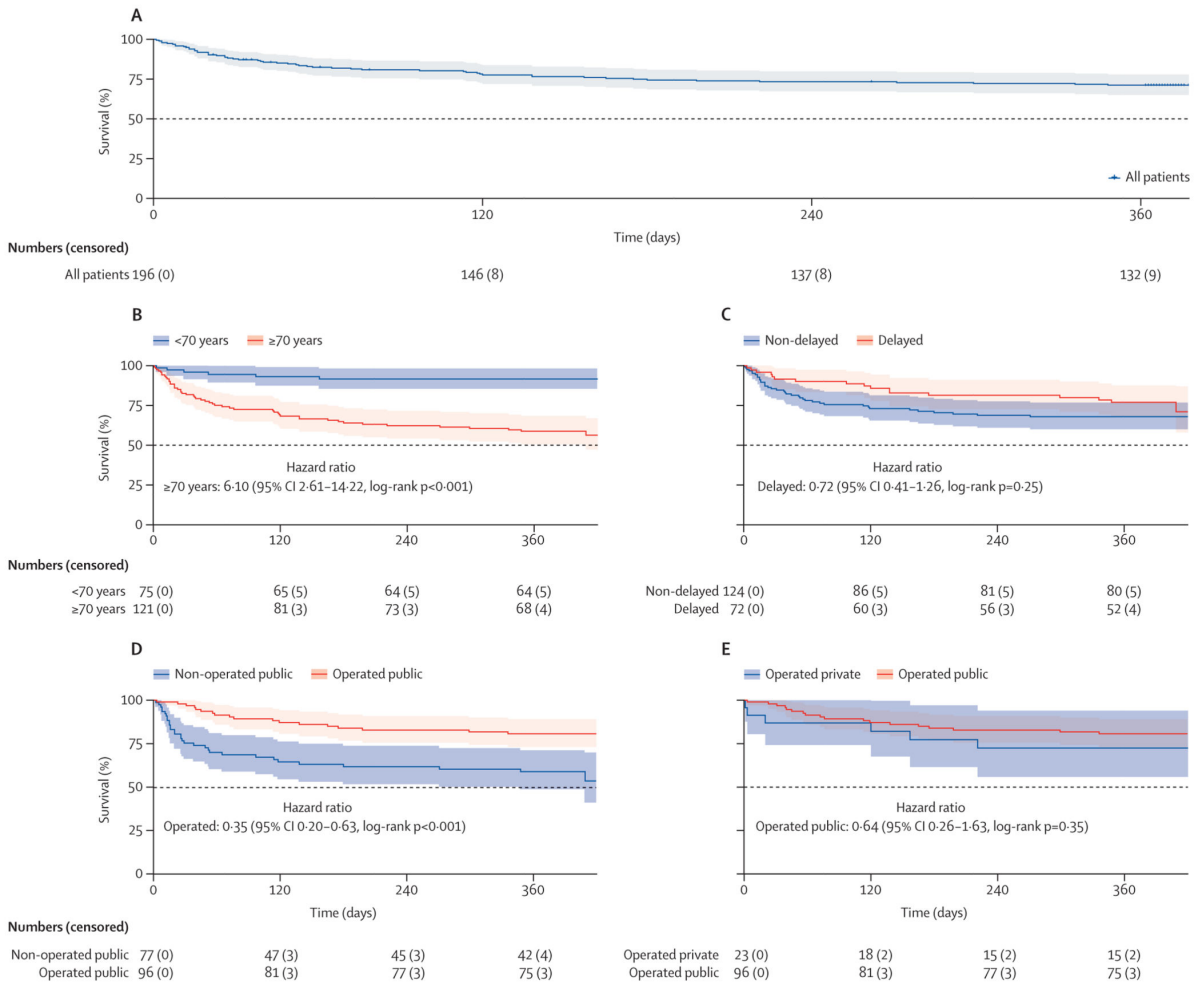
Kaplan–Meier survival function estimates over 12 months Data shown are estimates for patients overall (A), by age group (<70 years or ≥70 years; B), by delay to presentation (delayed >2 weeks or not delayed [≤ 2 weeks]; C), by operation status at public facilities (non-operated or operated; D), and by facility type in patients operated on (public or private facility; E). Global proportional hazard ratio test p values were p=0⋅41 for age, p=0⋅006 for delay to presentation, p=0⋅53 for operation status in public facilities, and p=0⋅67 for facility type in patients operated on. p values and examination of log-log residual plots indicate deviation from proportional hazard assumption for delay to presentation.

Overall, survival declined to 88% [95% CI 82–92] by 30 days and to 71% [64–77] by 12 months (figure 1A). Patients aged 70 years or older had lower survival probability than patients younger than 70 years at all timepoints (figure 1B). Although not significant, patients with delayed hospital presentation seemed to have marginally higher survival probabilities than those presenting within 2 weeks of injury (figure 1C); when stratified by sex, this difference was only apparent in women (appendix 2 p 6). For men, there was no difference in survival by presentation delay. By 12 months, only two (6%) of the 32 patients with highenergy trauma hip fractures had died, compared with 53 (34%) of 155 patients with low-energy trauma fractures. Survival probabilities were substantially higher in patients operated on at public and private facilities than in patients not operated on at public facilities (figure 1D, E).

**Figure 2:**
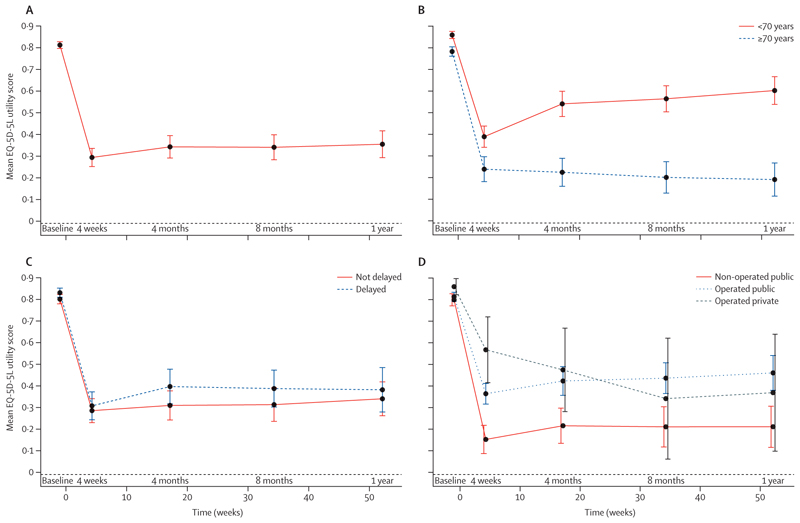
Mean EQ-5D-5L scores at sequential timepoints over 12 months after hip fracture Data shown are estimates for patients (A) overall, (B) by age group (<70 or ≥ 70 years), (C) by delay to presentation (delayed by >2 weeks or non-delayed [≤ 2 weeks]), and (D) by operation status (non-operated at public facilities or operated on at either public or private facilities). Baseline indicated recalled quality of life pre-injury; all other measures are prospective. EQ-5D-5L=5-level EQ-5D.

By 30-days post fracture, mean EQ-5D-5L utility score had fallen from a (recalled) pre-fracture score of 0⋅81 (95% CI 0⋅80–0⋅83) to a (prospective) score of 0⋅29 (0⋅25–0⋅34), indicating a sharp decline in quality of life. Minimal recovery was seen by 120 days (mean 0⋅34, 95% CI 0⋅29–0⋅39), then plateaued at a mean 0⋅35 (0⋅29–0⋅42) at 12 months (figure 2A and appendix 2 p 13). Sensitivity analyses restricted to those who survived the year (100 of whom completed 12-month follow-up) showed better recovery up to 12 months (mean utility score 0⋅60, 0⋅57–0⋅64; appendix 2 p 7). At all follow-up timepoints, recovery in HRQoL was substantially worse in those aged 70 years or older than in those who were younger (figure 2B); in the older group, HRQoL continued to fall throughout the year of follow-up (decline of 76% by 12 months), whereas, in the younger group recovery continued throughout the year to give a final utility score at 12 months 30% below baseline (appendix 2 p 13). HRQoL recovery did not vary by presentation delay (figure 2C). The lowest mean EQ-5D-5L utility score was seen at 30 days in patients admitted to a public hospital who did not receive an operation (mean 0⋅15, 0⋅09–0⋅22). For these patients, HRQoL barely recovered in the year of follow-up (figure 2D). Those operated on experienced fewer profound losses in HRQoL (figure 2D; appendix 2 p 13).

**Figure 3:**
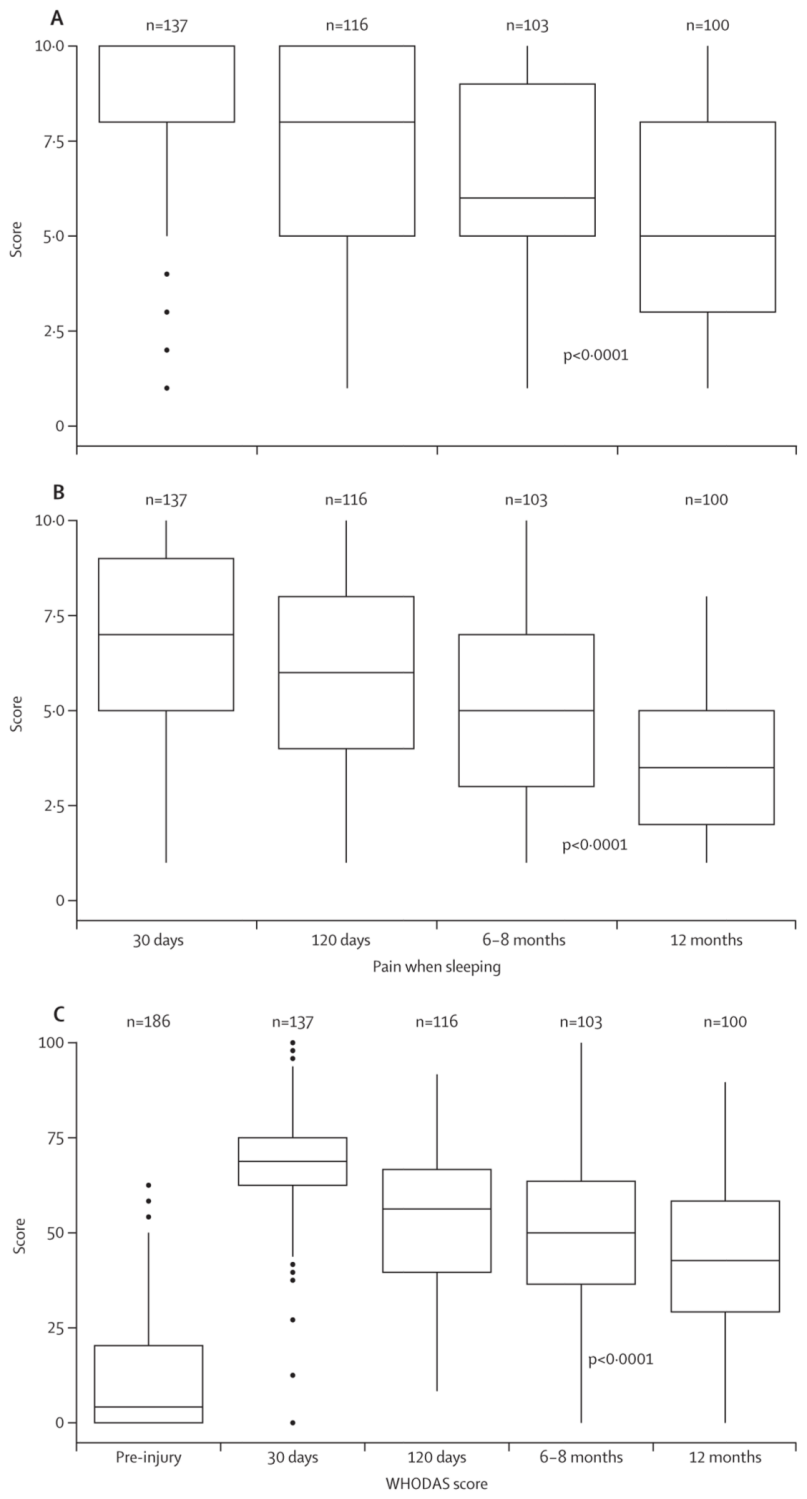
Median scores for pain interfering with ability to walk (A), sleep (B), and WHODAS (C) at sequential timepoints over 12 months after hip fracture Box and whisker plots displaying median (IQR) and range of values. WHODAS=WHO Disability Assessment Schedule version 2.0.

Of those alive at 30 days and able to provide data (n=137), only four (3%) had completely resolved hip pain, whereas the remainder 133 (97%) had ongoing pain from their hip injury (23 [17%] describing this as “all the time every day”; appendix 2 p 14). By 12 months, of 100 patients alive and able to provide data, 97 (97%) still reported pain from their hip injury. At 30 days, 135 (99%) patients experienced pain that interfered with their ability to walk (median pain severity score 10, IQR 8–10); by 12 months, 94 (94%) still had pain that interfered with walking, although the median severity score had lessened (5, 3–8; figure 3A). Furthermore, by 12 months, patients who received operative management had a little less pain that interfered with walking (median pain severity score 5, IQR 3–8) than those who had not had an operation (median score 6, 5–9; appendix 2 p 8). Pain interfering with sleep was also common, with 80 (80%) patients still experiencing pain that interfered with sleep at 12 months. The median severity scores for pain that interfered with sleep were 7 (IQR 5–9) at 30 days and 3⋅5 (2–5) at 12 months in the population overall (figure 3B). At 12 months, those who had had an operation had less pain interfering with sleep (median severity score 3, 2–5) than those who had not had an operation (4, 3–7; appendix 2 p 8).

Pre-injury disability was common; 119 (64%) had a WHODAS ≥1 (of whom 107 [58%] had emotional health problems, and 72 [39%] had difficulties walking for long distances). However, the median WHODAS score rose from 4⋅2 (IQR 0⋅0–20⋅8) pre-injury to 68⋅8 (62⋅5–75⋅0) at 30 days (in the 137 surviving patients with available data; figure 3C); at this point in time, 132 (96%) had a WHODAS score of 1 or higher, indicating disability (appendix 2 p 14). In the overall population, disability severity gradually reduced to a median WHODAS score of 42⋅7 (IQR 29⋅2–58⋅3) by 12 months, by which time only 2% were disability-free (WHODAS score=0). Those who had had an operation saw a little more improvement in disability by 12 months, with a median score of 39⋅6 (22⋅9–54⋅2), than did those who were not managed operatively (median score 45⋅8, 37⋅5–66⋅6; appendix 2 p 8).

## Discussion

This study has shown that individuals in Zimbabwe experience profound losses in length and quality of life after hip fracture. Nearly three in ten patients had died by 12 months of follow-up. Despite international standards recommending early surgery for hip fracture to restore mobility and alleviate pain,^34^ non-surgical management is more common in Zimbabwe than previously recognised (39⋅3%),^15^ and it is associated with markedly higher mortality and worse quality of life than surgical management. In the year after a hip fracture, we further observed evidence of long-term pain and disability. Patients treated non-surgically were characterised by markers of deprivation (eg, lower household income, malnutrition, and lower educational attainment), rather than older age, suggesting it is primarily a patient’s ability to pay that determines management.

Despite including a relatively young population with hip fracture in this study (median age 74 years), we observed a high mortality of 14% at 30 days. This 30-day mortality is more than double that reported in high-income countries such as the UK and the USA (6%).^35,36^ Zimbabwean patients aged 70 years or older had a particularly high mortality at 1 year (42%). These findings align with those observed in neighbouring South Africa, where mortality rates following hip fracture were 13% at 1 month and 33⋅5% at 12 months.^12^ A single-centre study in Ghana, involving 76 participants older than 50 years, reported lower mortality rates at 1 month (6⋅6%) and 12 months (19⋅7%).^11^

We observed better survival probabilities in female patients with delayed hospital presentation than in those presenting within 2 weeks of injury. These findings might be due to the fact that those who are healthier and fitter could be more able to cope with their injury and therefore take longer to present, or to the fact that some of those with poorer premorbid conditions might have died before reaching hospital. These hypotheses might also explain why HRQoL recovery did not vary by presentation delay.

The very long lengths of hospital stay in public hospitals, for patients both operated on and not operated on, might indicate the length of time it takes for a patient’s family to mobilise the out-of-pocket costs necessary to facilitate surgery. Such delays would be expected to increase risk of complications, such as pressure sores and venous thromboembolic disease, as well as health-care costs, which is an area that requires further research. In contrast, patients in the private sector experienced longer hospital stays after their operation (~ 6 days) than did those following an operation in public hospital (~ 3 days), suggesting potentially more access to active rehabilitation in private but not public facilities.

Results demonstrated clinically important adverse impacts of hip fracture on HRQoL for all patients, but particularly for those older than 70 years and for those not given an operative fixation. These findings align with data from high-income countries, which showed that older patients with hip fracture, especially those older than 80 years, also exhibit poor HRQoL recovery, indicating the vulnerability of this population to poor post-fracture outcomes.^8^ For context, our study population had much lower EQ-5D utility scores at 12 months (mean 0⋅35, 95% CI 0⋅29–0⋅41) than the EQ-5D utility scores reported in patients after a stroke in Sierra Leone (median 0⋅76, 95% CI 0⋅47–1⋅0),^37^ highlighting the severe impact of hip fracture. Importantly, patients who were operated on experienced less severe HRQoL trajectories, further emphasising the importance of access to surgical intervention.

Both pain and disability are key drivers of HRQoL. The limited recovery of functional ability highlights the broader impact of hip injuries. The persistent and severe disability observed a year after the hip fracture, with only 2% of patients regaining their pre-injury disability-free status, indicates a substantial long-term burden on the patient, relatives, and health-care systems. Persistent pain was still present a year later for the majority of patients. These findings likely reflect the near absence of post-fracture community rehabilitation, and widespread stockouts of basic analgesia in Zimbabwe.

Zimbabwe spends just 2⋅8% of its gross domestic product on health expenditure,^38^ and very few people have the privilege of medical aid cover. Our findings point to critical areas for quality improvement in hip fracture management. Clinical guidelines to standardise care, such as time to surgery, analgesia, and postoperative rehabilitation, are urgently needed, complemented by health professional educational and training events. Financial schemes are needed to enable timely emergency surgery.

The strengths of this study were the inclusion of both public and private facilities, a 1-year follow-up with relatively low loss to follow-up for this setting and this population, and a focus on patient outcomes. Standardised classification of hip fractures was used with external verification. Limitations of this study included self-reported HIV status, which might have been underreported due to stigma, affecting understanding of the relationship of HIV to the study outcomes. Although delayed presentation was common, it was not associated with lower survival as would have been expected,^35^ suggesting a healthy survivor bias in those with delayed presentation who made it to a hospital. Not all patients had an x-ray-confirmed fracture; however, in anticipation of this issue, the study design planned for a clinical diagnosis when a radiograph was not available. The Cox proportional hazards analyses might have been underpowered, rendering the analyses exploratory, reducing the ability to detect associations with mortality.

Adverse health outcomes in patients with hip fracture in Zimbabwe, particularly among older adults and those treated non-surgically, require the implementation of national guidelines that prioritise timely and equitable surgical care. Clinical profiles rather than financial status should dictate care pathways. Future interventions must focus on reducing delays in operative management and, by implication, reducing health costs associated with long hospital stays. Furthermore, expanding access to rehabilitation, and addressing socioeconomic barriers to care, are crucial to improving survival, functional recovery, and quality of life in this population. Finally, these findings underscore the urgent need for clinical guidelines to standardise care delivery, and targeted interventions to improve equitable access to care, to improve outcomes for patients with hip fractures in this region.

## Supplementary Material

Supplementary appendix 1

Supplementary appendix 2

## Figures and Tables

**Table 1:** Baseline characteristics of the study population overall and stratified by age and facility type or operation status

	Total (n=196)	Age		p value*	Facility type and operation status			p value*
		<70 years (n=75)	>70 years (n=121)		Not operated, public facility (n=77)	Operated, public facility (n=96)	Operated, private facility (n=23)	
**Consent**								
Consented	190/196 (97%)	73/75 (97%)	117/121 (97%)	··	74/77 (96%)	96/96 (100%)	20/23 (87%)	··
Died before providing consent	6/196 (3%)	2/75 (3%)	4/121 (3%)	··	3/77 (4%)	0	3/23 (13%)	··
**Patient characteristics**								
Median age, years (IQR)†	74 (62·5–83)	57 (50–65)	81 (75–86)	<0·0001	74 (66–83)	71·5 (58–82)	79 (69–85)	0·12
Sex								0·930
Female†	96/196 (49%)	22/75 (29%)	74/121 (61%)	<0·0001	39/77 (51%)	46/96 (48%)	11/23 (48%)	··
Male†	100/196 (51%)	53/75 (71%)	47/121 (39%)	··	38/77 (49%)	50/96 (52%)	12/23 (52%)	··
Harare resident†	106/196 (54%)	47/75 (63%)	59/121 (49%)	0·06	43/77 (56%)	46/96 (48%)	17/23 (74%)	0·07
Black African	181/190 (95%)	70/73 (96%)	111/117 (95%)	0·75	74/74 (100%)	93/96 (97%)	14/20 (70%)	<0·0001
Educational attainment‡				<0·0001				<0·0001
None	21/190 (11%)	7/73 (10%)	14/117 (12%)	··	8/74 (11%)	13/96 (14%)	0	··
Primary	95/190 (50%)	19/73 (26%)	76/117 (65%)	··	42/74 (57%)	47/96 (49%)	6/20 (30%)	··
Secondary	46/190 (24%)	35/73 (48%)	11/117 (9%)	··	15/74 (20%)	25/96 (26%)	6/20 (30%)	··
Post-secondary	17/190 (9%)	9/73 (12%)	8/117 (7%)	··	0	9/96 (9%)	8/20 (40%)	··
Marital status‡				<0·0001				0·10
Married or cohabiting	82/190 (43%)	47/73 (64%)	35/117 (30%)	··	27/74 (36%)	44/96 (46%)	11/20 (55%)	··
Separated, divorced, or never married	22/190 (12%)	15/73 (21%)	7/117 (6%)	··	12/77 (16%)	7/96 (7%)	3/20 (15%)	··
Widowed	82/190 (43%)	10/73 (14%)	72/117 (62%)	··	33/74 (45%)	43/96 (45%)	6/20 (30%)	··
Residence type‡				0·10				0·12
Own home	156/190 (82%)	59/73 (81%)	97/117 (83%)	··	62/74 (84%)	77/96 (80%)	17/20 (85%)	··
Relatives home	24/190 (13%)	10/73 (14%)	14/117 (12%)	··	8/74 (11%)	16/96 (17%)	0	··
Residential or nursing home	4/190 (2%)	0	4/117 (3%)	··	1/74 (1%)	2/96 (2%)	1/20 (5%)	··
Other	5/190 (3%)	4/73 (5%)	1/117 (1%)	··	2/74 (3%)	1/96 (1%)	2/20 (10%)	··
Household income ≤US$100‡	115/190 (61%)	40/73 (55%)	75/117 (64%)	0·06	55/74 (74%)	57/96 (59%)	3/20 (15%)	<0·0001
Current tobacco smoker	31/190 (16%)	19/73 (26%)	12/117 (10%)	0·004	11/74 (15%)	18/96 (19%)	2/20 (10%)	0·57
Consumes alcohol‡	51/190 (27%)	31/73 (42%)	20/117 (17%)	0·0004	17/74 (23%)	29/96 (30%)	5/20 (25%)	0·39
Living with HIV (self-reported) ‡	24/190 (13%)	20/73 (27%)	4/117 (3%)	<0·0001	8/74 (11%)	16/96 (17%)	0	<0·0001
Low MUAC (≤23 cm) ‡	40/190 (21%)	10/73 (14%)	30/117 (26%)	0·02	23/74 (31%)	17/96 (18%)	0	0·001
**Hip fracture specific characteristics**								
Hip fracture type† ‡				0·35				0·15
Intracapsular	82/196 (42%)	36/75 (48%)	46/121 (38%)	··	34/77 (44%)	36/96 (38%)	12/23 (52%)	··
Intertrochanteric	79/196 (40%)	26/75 (35%)	53/121 (44%)	··	31/77 (40%)	43/96 (45%)	5/23 (22%)	··
Subtrochanteric	27/196 (14%)	11/75 (15%)	16/121 (13%)	··	6/77 (8%)	17/96 (18%)	4/23 (17%)	··
Mechanism of injury indicates low-energy trauma†	162/196 (83%)	46/75 (61%)	116/121 (96%)	<0·0001	65/77 (84%)	76/96 (79%)	21/23 (91%)	0·34
Presentation delayed by >2 weeks from injury†	72/196 (37%)	33/75 (44%)	39/121 (32%)	0·10	31/77 (40%)	37/96 (39%)	4/23 (17%)	0·12
Presenting to a public hospital†	173/196 (88%)	69/75 (92%)	104/121 (86%)	0·20	77/77 (100%)	96/96 (100%)	0	<0·0001
Operated†	119/196 (61%)	48/75 (64%)	71/121 (59%)	0·46	0	96/96 (100%)	23/23 (100%)	<0·0001
ASA grade‡				0·10				<0·0001
I Normal health	27/190 (14%)	16/73 (22%)	11/117 (9%)	··	0	20/96 (21%)	7/20 (35%)	··
II Mild systemic disease	69/190 (36%)	25/73 (34%)	44/117 (38%)	··	0	58/96 (60%)	11/20 (55%)	··
III Severe systemic disease	15/190 (8%)	4/73 (5%)	11/117 (9%)	··	0	15/96 (16%)	0	··
Median hospital length of stay in days (IQR)	22 (13–32)	24 (14–35)	21 (12–32)	0·16	24 (16–33)	24 (13–35)	10·5 (8–13)	0·0001
Median hospital days until surgery (IQR) ‡	16 (8–27)	16 (9–29)	16 (7–27)	0·72	NA	20 (10–30)	4 (2–7·5)	0·0001
Median hospital days from surgery until discharge (IQR) ‡	3 (2–6)	3 (2–5)	3 (2–6)	0·63	NA	3 (2–5)	5·5 (3·5–8)	0·003

Data are n/N (%), unless otherwise specified. MUAC=mid-upper arm circumference. ASA=American Society of Anesthesiologists.

* χ ^2^ p value shown for categorical variables and Kruskal–Wallis p value for comparison of medians.

† Variables available for all 196 with a minimum dataset, including those who consented and those who died before providing consent (other variables were only collected for the patients who provided consent; n=190).

‡ Missing values: educational level n=11; marital status n=4; residence type n=1; household income n=10; alcohol intake n=2; HIV n=47; MUAC n=29; hip fracture type n=8; ASA grade n=79; date of surgery n=1

**Table 2:** Patient and fracture characteristics associated with the odds of having an operative fixation for hip fracture in Zimbabwe

	All (n=196)*	Operated (n=119)	Odds ratio (95% CI)	p value
**Patient characteristics**				
Age <70 years	75	48 (64%)	1 (ref)	
Age ≥70 years	121	71 (59%)	0·80 (0·44–1·44)	0·46
Male	100	62 (62%)	1 (ref)	··
Female	96	57 (59%)	0·89 (0·50–1·59)	0·70
Higher educationt	84	48 (76%)	1 (ref)	··
Primary or lesst	116	66 (57%)	0·41 (0·21–0·82)	0·009
Household income <$100/montht	115	60 (52%)	1 (ref)	··
Household income >$100/montht	65	50 (77%)	3·06 (1·54–6·05)	0·001
Non-smoker	159	99(60%)	1 (ref)	··
Current tobacco smoker	31	20 (65%)	1·29 (0·54–2·66)	0·66
No alcohol consumedt	137	82(60%)	1 (ref)	··
Alcohol consumptiont	51	34 (67%)	1·34 (0·68–2·63)	0·39
MUAC >23 cmt	121	85 (70%)	1 (ref)	··
MUAC ≤23 cmt	40	17 (43%)	0·31 (0·15–0·65)	0·002
No HIV self-reportedt	119	73 (61%)	1 (ref)	··
HIV self-reportedt	24	16(67%)	1·26 (0·50–3·18)	0·62
**Hip fracture specific characteristics**				
Presentation ≤2 weeks from injury	124	78 (63%)	1 (ref)	··
Presentation >2 weeks from injury	72	41 (57%)	0·78 (0·43–1·41)	0·41
Intracapsular fracture	82	48 (59%)	1 (ref)	··
Intertrochanteric fracture	79	48(61%)	1·10 (0·58–2·06)	0·77
Subtrochanteric fracture	27	21 (78%)	2·48 (0·90–6·79)	0·08
High-energy trauma	34	22 (65%)	1 (ref)	··
Low-energy trauma	162	97(60%)	0·81 (0·38–1·76)	0·60
Presenting to private facility	23	23 (100%)	··	··
Presenting to public facility	173	96 (55%)	··	··

Data are n (%), unless otherwise specified. MUAC=mid-upper arm circumference.

* Variables available for all 196 with a minimum dataset including those who consented and those who died before providing consent (other variables were only collected for the patients who provided consent; n=190).

† Missing values: educational level n=11; household income n=10; alcohol intake n=2; MUAC n=29; HIV n=47; hip fracture type n=8.

## Data Availability

Data are available upon reasonable request. Researchers can access participant level data via data.bris, provided ethical approvals are in place.

## Research in context

### Evidence before this study

For older people, fragility fractures of the hip are a common and devasting injury, causing severe pain, loss of mobility, and sometimes death. The international standard of care is an operation to fix the fracture, alleviate pain, and restore function. Postoperative rehabilitation is crucial to maximise recovery and promote independence. Africa has one of the most rapidly ageing populations, yet hip fracture outcomes in this region are poorly understood, and management is mixed.

We searched PubMed on Oct 8, 2024, for articles in English published in the past 20 years, using the MeSH terms ((hip fractures[MeSH Terms])) AND (africa south of the sahara[MeSH Terms]). This search returned 38 studies. Only three studies from across the African continent (two from South Africa and one from Ghana) reported mortality and select clinical and radiological outcomes in patients with hip fractures. No patient-reported outcomes were studied (eg, quality of life, pain), nor did any previous study address variation in care between care facilities. Although one study in South Africa reported higher 1-month and 1-year mortality following hip fracture than mortality reported in high-income countries, another study in South Africa with about 14% loss to follow-up found lower 1-year mortality rates. The study in Ghana, a small single-centre study (N=76) that mainly focused on predictors of mortality following hip fracture, reported lower mortality than that seen in high-income countries.

### Added value of this study

This study provides a novel evaluation of mortality, health-related quality of life, pain, and disability in the year following hip fracture, including a comparative analysis of patients treated in public versus private health-care facilities in Zimbabwe. The study identified high early mortality rates, profound loss of health-related quality of life, persistent pain through to a year, and almost universal chronic disability following hip fracture. Contrary to international standards, 39% of patients did not receive an operation to fix their fracture; this group had lower quality of life and were more likely to have died by 1 year than patients who were operated on. This study identifies several key areas for improvement of hip fracture care.

### Implications of all the available evidence

This study highlights substantial health inequities in the provision of hip fracture care. Inadequate care is leading to high levels of disability, which are likely to have a long-term impact on household productivity. National clinical guidelines and standards of care need to be implemented urgently, to prioritise timely and equitable surgical care, ensuring that clinical profiles rather than financial status dictate care pathways. Future interventions must focus on reducing delays in operative management, expanding access to rehabilitation, and addressing socioeconomic barriers to care, which are crucial to improving survival, functional recovery, and quality of life in this population, as well as being likely to confer health-care savings from reduced bed occupancy.
