# Supplementary material for: Impact of hip fracture on survival, disability, pain, and health-related quality of life in Zimbabwe: a prospective cohort study
Source: Lancet Healthy Longev. Author manuscript; Available in PMC 2026 Feb 19. (PMC7618745; doi:10.1016/j.lanhl.2025.100766)
Supplement: Supplementary appendix 1 [file EMS212159-supplement-Supplementary_appendix_1.pdf]

THE LANCET
Healthy Longevity

Supplementary appendix 1

This translation in Shona was submitted by the authors and we reproduce it as supplied. It has not been peer reviewed. *The Lancet*'s editorial processes have only been applied to the original in English, which should serve as reference for this manuscript.

Shanduro iyi muShona yakatumirwa nevanyori uye tinoiburitsa sekupihwa kwatakaitwa. Huye hayina kuongororwa nevezera. Maitiro ekupepeta e *Lancet* angoshandiswa chete kugwaro rekutanga, iro rinofanira kushanda seumboo hwechinyorwa

Supplement to: Nasser MI, Burton A, Wilson H, et al. Impact of hip fracture on survival, disability, pain, and health-related quality of life in Zimbabwe: a prospective cohort study. *Lancet Healthy Longev* 2025. https://doi.org/10.1016/j.lanhl.2025.100766

Impact of hip fracture on survival, disability, pain, and health-related quality of life in Zimbabwe: a prospective cohort study

Pfupiso

Hwaro: Ruzhinji rwevanhu muAfrica vari kurarama kwenguva yakareba, uye njodzi dzekutyoka/kuvhunika dziri mabhonzo dziri kuwedzera. Takaita ongororo munguva yakaita gore rimwe chete pavanhu vakura vakambotyoka muhudyu muZimbabwe.

Nzira yakashandiswa kuita tsvakurudzo: Mutsvakurudzo ino yaitevera boka revakwegura vane makore makumi mana kana kupfuura mushure mekunge vachangobva mukupiwa mubhedha muchipatara vakaita njodzi yekutyoka bhonzo remuhudyu, muHarare (muzvipatara zviviri zveruzhinji uye zvishanu zvinoshanda zvakazvimirira zvoga) kubva musiwa 15 Gumiguru 2021 kusvika musiwa 14 Gumiguru 2022 uye vakateverwa nevavashandi vetsvakurudzo kwemwedzi gumi nemiviri. Chinangwa chikuru chetsvakurudzo chaiva chekuda kuona kuti vangani vairarama mushure mekusangana nenjodzi yekutyoka muhudyu uye takatarisa izvi tichishandisa inonzi Kaplan-Meier curves munguva dzakasiyana (mushure memazuva makumi matatu [30 days], mazuva zana nemakumi maviri [120 days], mwedzi mitanhatu kusvika misere [6–8 months], uye mwedzi gumi nemiviri [12 months] kubva pakupinda mutsvakurudzo). Ongororo iyi yakaitwa tichitarisa vanhu vese vakapinda mutsvakurudzo uye takaisa vanhu mumapoka zvichienderana nemakore avo (vasati vasvitsa makore makumi manomwe [<70] tichienzanisa nevane makore makumi manomwe zvichikwira [>70], uye vakanonoka kuenda kuchipatara nevasina (vasina kunonoka [nguva isingadariki masvondo maviri] zvichienzaniswa nevakanonoka [vakadarika masvondo mavirir vasati vaenda kuchipatara]). Ongororo yakatarisawo marapirwo evanhu vakatyoka muhudyu uye kuti chipatara cheruzhinji here kana chinoshanda chakazvimirira chega, uye marapirwe akaitwa acho kuti munhu akaoparetwa here kana haana, Tsvakurudzo yakatarisawo huwandu hwevanhu vane hutano hwakanaka pakurarama kwavo (HRQoL) tichishandisa nzira yematanho mashanu pachiringu inozivikanwa nezita rekuti EQ-5D (EQ-SD-5L), tichitarisa marwadzo emuhudyu vanhu vachizvitaurira vega. Takashandisa chikero chekubva pazero (Hapana marwadzo) kusvika pashanu (5) kuva nemarwadzo nguva dzese, pachitarisa kuti zvaivatadzisa kurara nekufamba zvakanaka here 1 zvichireva kuti hazvitadzise kufamba kana kurara kusvika ku10 kureva kuti zvaivatadzisa kurara kana kufamba zvachose. Uye ongororo yemarwadzo pamwechete nehurema zvichiiswa pachikero chinodaidzwa kuti WHO disability Assessment schedule version 2.0 (WHODAS).

Zvakabuda mutsvakurudzo: Pavarwere zana nemakumi mapfumbamwe nematanhatu (196) vaiva vakatyoka hudyu, makumi mapfumbamwe nematanhatu (96) vanova zvikamu zvina nepfumbamwe kubva muzana (49%) vaiva vanhukadzi uye vanhu zana (100) vanova zvikamu makumi mashanu nechimwe (51%) vanhurume, makore evanhu ava aiva makumi manomwe nemana (74 years; IQR 62.5–83), Vanhu zana nemakumi matanhatu nevaviri (162) vakaonekwa kuti vakanga vamboita inonzi pachirungu fragility fracture (Kutyoka bhonzo pachishandiswa simba shoma). Pamwechete, vanhu zana nemakumi manomwe nevatatu (173) (zvikamu makumi masere nevasere [88%] kubva muzana) vakanga vachionekwa pazvipatara zveruzhinji, uye makumi mapfumbamwe nevatanhatu (96) (zvikamu makumi mashanu nevashanu [55%] kubva muzana) yavo vakaitwa opareshenìyekubatanidza bhonzo ravo muhudyu. Iyo inozivikanwa muchirungu nekuti operative hip fixation. Zvichienzaniswa, pavanhu makumi maviri nevatatu (23) (zvikamu gumi nezviviri kubva muzana [12%]) avo vakanga vachirapirwa muzvipatara zvinoshanda zvakazvimirira zvega vose

vakaitwa oparesheni iyi. Mushure memwedzi gumi nemiviri 12), vanhu makumi mashanu nevashanu (55) (makumi maviri nevapfumbamwe kubva muzana [29%]) vakanga vafa (makumi mana nevapfumbabwe [49]) (makumi mana nevaviri kubva muzana [42%]) pavanhu zana negumi nevanomwe (117) vaive nemakore makumi manomwe zvichikwira (>70) uye vanhu vatanhatu (6) (zvikamu zvipfumbamwe kubva muzana [9%] vevanhu makumi manomwe [70] vaiva nemakore makumi manomwe [70] zvichidzika). Muzvipatara zveruzhinji, vanhu makumi matatu nemumwe (31) (zvikamu makumi mana nezviviri kubva muzana [42%]) yevanhu makumi manomwe nevatatu (73) pavarwere vasina kuoparetwa vakafa tichienzanisa nevanhu gumi nevasere (18) (zvikamu gumi nevapfumbamwe kubva muzana [19%]) pavanhu makumi mapfumbamwe nevatatu (93) vakaitwa oparesheni. Pamwechete, mikana yekurarama yakaderera kusvika pazvikamu makumi masere nemasere kubva muzana (88%) (95 CI 82–92) mushure memazuva makumi matatu (30) uye kusvika kuzvikamu makumi manomwe nechimwe kubva muzana (71%) (64-77) mushure memwedzi gumi nemiviri (12). Mikana yekurarama yakanga iri mishoma pavanhu vemakore makumi manomwe zvichikwira (70 years and above) tichienzanisa neavo vakanga vasati vasvika makore makumi manomwe (<70) (mortality hazard ratio [HR] for ≥70 years 6·10, 95% CI 2·61−14·22). Mean HRQoL utility score yakadzika kubva pa0.81 (95% CI 0·80– 0·83) munhu asati atyoka kusvika pa 0·29 (0·25–0·34) pamazuva makumi matatu kubva munhu paakasangana nedambudziko rekutyoka bhonzo remuhudyu. Varwere vakaratidza kupora kushomasa mushure mamazuva zana nemakumi maviri (120 days) (0·34,0·29–0·39). Mushure memwedzi gumi nemiviri (12), zvikamu makumi mapfumbamwe nezvinomwe kubva muzana (97%) vevanhu zana vapenyu vaikwanisa kuzvidairira vaiti vakanga vachiri kunzwa marwadzo kubva pakukuvara kwavakanga vaita muhudyu. Hurema hwekubva pakutyoka bhonzo remuhudyu hwakanga hwakada kuenderana muboka iri apo viri chete (zvikamu zviviri kubva muzana [2%] pavanhu zana vakanga vasina hurema (WHODAS=0) mushure memwedzi gumi nemiviri.

Tsanangudzo: Kubva pakutyoka bhonzo remuhudyu, mukana wekurarama uye kunaka kwemararamiro kwakadzika zvakanyanya pavanhu vakapinda mutsvakurudzo ino. Zvakawanikwa izvi mutsvakurudzo ino zvinoburitsa pachena kuti gwaro rinotsanangura mabatirwo nemarapirwo evanhu vanenge vatyoka mabhonzo emuhudyu rinofanirwa kushandiswa senzira yekuvandudza mabatirwo nemarapirwo edambudziko rekutyoka mabhonzo muhudyu sezvo dambudziko iri riri kufungidzirwa kuti riri kukura mudunhu rino reAfrica.

Rutsigiro rwemari: Wellcome Trust
