# Supplementary material for: Impact of hip fracture on survival, disability, pain, and health-related quality of life in Zimbabwe: a prospective cohort study
Source: Lancet Healthy Longev. Author manuscript; Available in PMC 2026 Feb 19. (PMC7618745; doi:10.1016/j.lanhl.2025.100766)
Supplement: Supplementary appendix 2 [file EMS212159-supplement-Supplementary_appendix_2.pdf]

THE LANCET
Healthy Longevity

Supplementary appendix 2

This appendix formed part of the original submission and has been peer reviewed.
We post it as supplied by the authors.

Supplement to: Nasser MI, Burton A, Wilson H, et al. Impact of hip fracture on survival, disability, pain, and health-related quality of life in Zimbabwe: a prospective cohort study. *Lancet Healthy Longev* 2025. https://doi.org/10.1016/j.lanhl.2025.100766

Supplementary Materials

Contents

Supplementary Information S1 Ethical and governance permissions.. 2

Supplementary Figure S1. Consort diagram of the study population.. 3

Supplementary Figure S2. Kaplan-Meier survival function estimates over 12-months for males and females.. 4

Supplementary Figure S3. Crude (panel A) and age-adjusted (panel B) hazard ratios (and 95%CI) of 1-year mortality risk following hip fracture 5

Supplementary Figure S4. Kaplan-Meier survival function estimates over 12-months for patients delayed (> 2weeks) vs not delayed (≤2weeks) for males (panel A) and females (panel B). 6

Supplementary Figure S5. Mean changes in EQ-5D-5L over 12-months for all patients, excluding 54 who died. 7

Supplementary Figure S6. Median scores for pain interfering with ability to walk in those operated (panel A) and not operated (panel B), pain interfering with ability to sleep in those operated (panel C) and not operated (panel D) and WHODAS in those operated (panel E) and not operated (panel F), at 30-days, 120-days, 6-8 months and 12-months following hip fracture 8

Supplementary Table S1. Baseline characteristics of the study population stratified by consent status 9

Supplementary Table S2. Baseline characteristics of the study population stratified by whether presentation delayed (>2 weeks after injury).. 10

Supplementary Table S3. Baseline characteristics of the study population stratified by the reporting of vital status. 11

Supplementary Table S4. Cumulative mortality over 12-months, stratified by age (<70 vs. ≥70 years), delayed presentation (>2 weeks), and facility type and operation status. 12

Supplementary Table S5: Mean change in EQ-5D-5L at 30, 120days and 12-months overall, and stratified by age, delayed presentation, and by facility type and operation status 13

Supplementary Table S6: Pain and disability following hip fracture 14

Supplementary Information S1 Ethical and governance permissions

Ethical and governance approvals were obtained from: The Medical Research Council of Zimbabwe (14/07/2021 ref MRCZ/A/2706); Biomedical Research and Training Institute (19/02/2021 ref AP161/2021); Sally Mugabe Central Hospital (29/01/2021 ref HCHEC/250121/06); University of Zimbabwe College of Health Sciences and the Parirenyatwa group of hospitals (25/02/2021); Harare City Health (27/01/2021); Research Council of Zimbabwe (RCZ, 14/07/2021 refs 04246 and 04248).

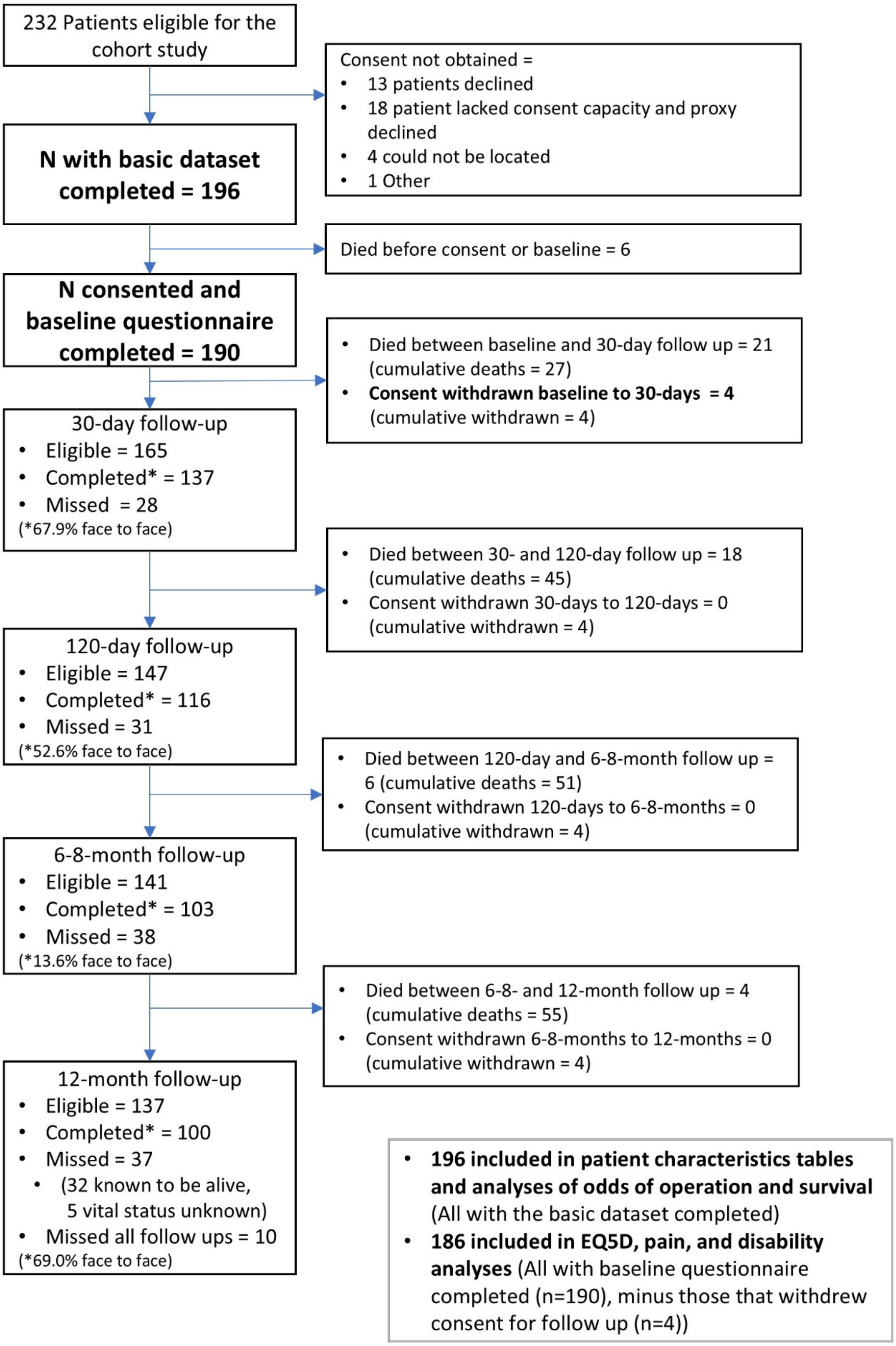

Supplementary Figure S1. Consort diagram of the study population.

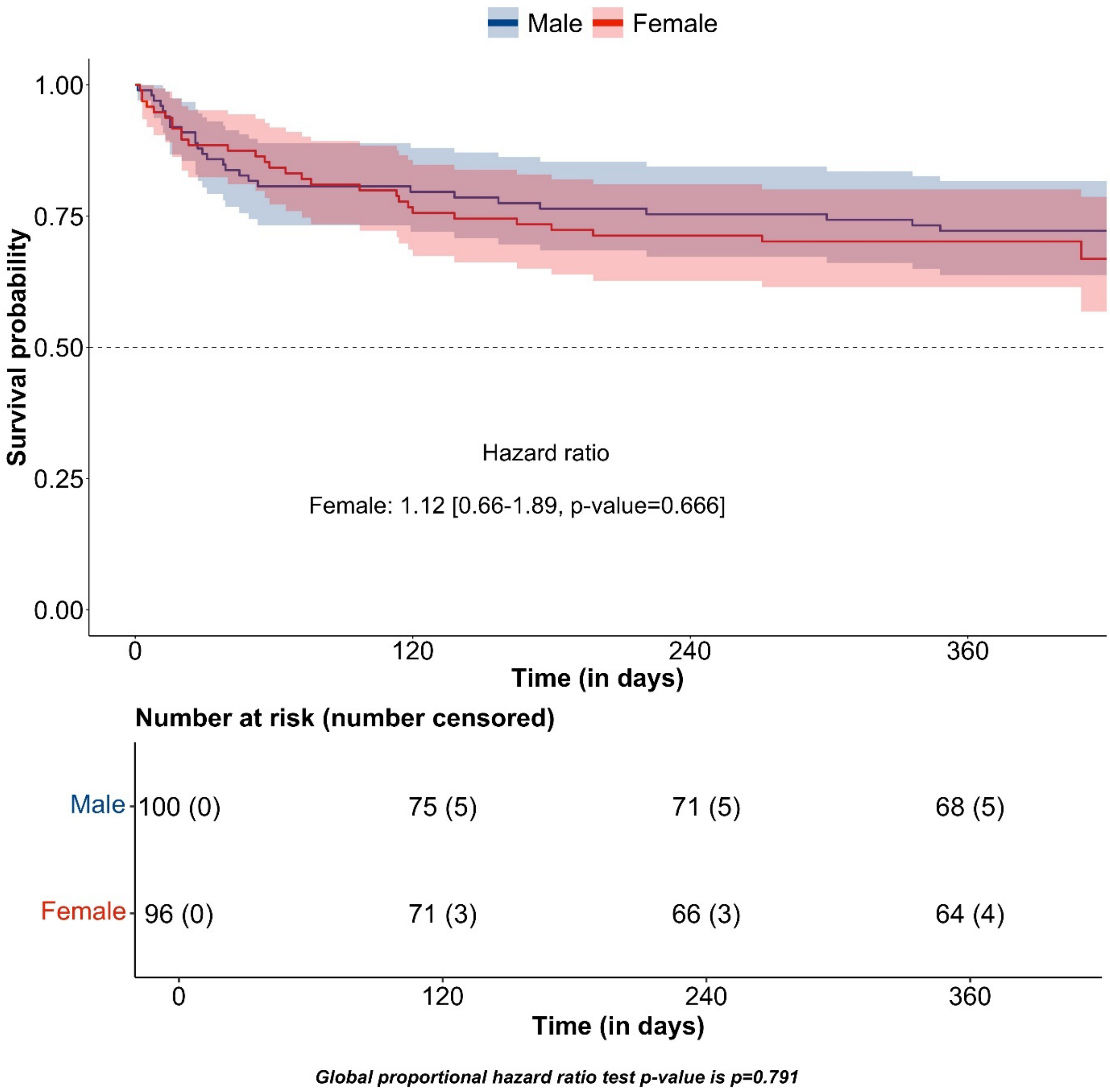

Supplementary Figure S2. Kaplan-Meier survival function estimates over 12-months for males and females. Proportionality assumptions were checked and confirmed assumptions were upheld.

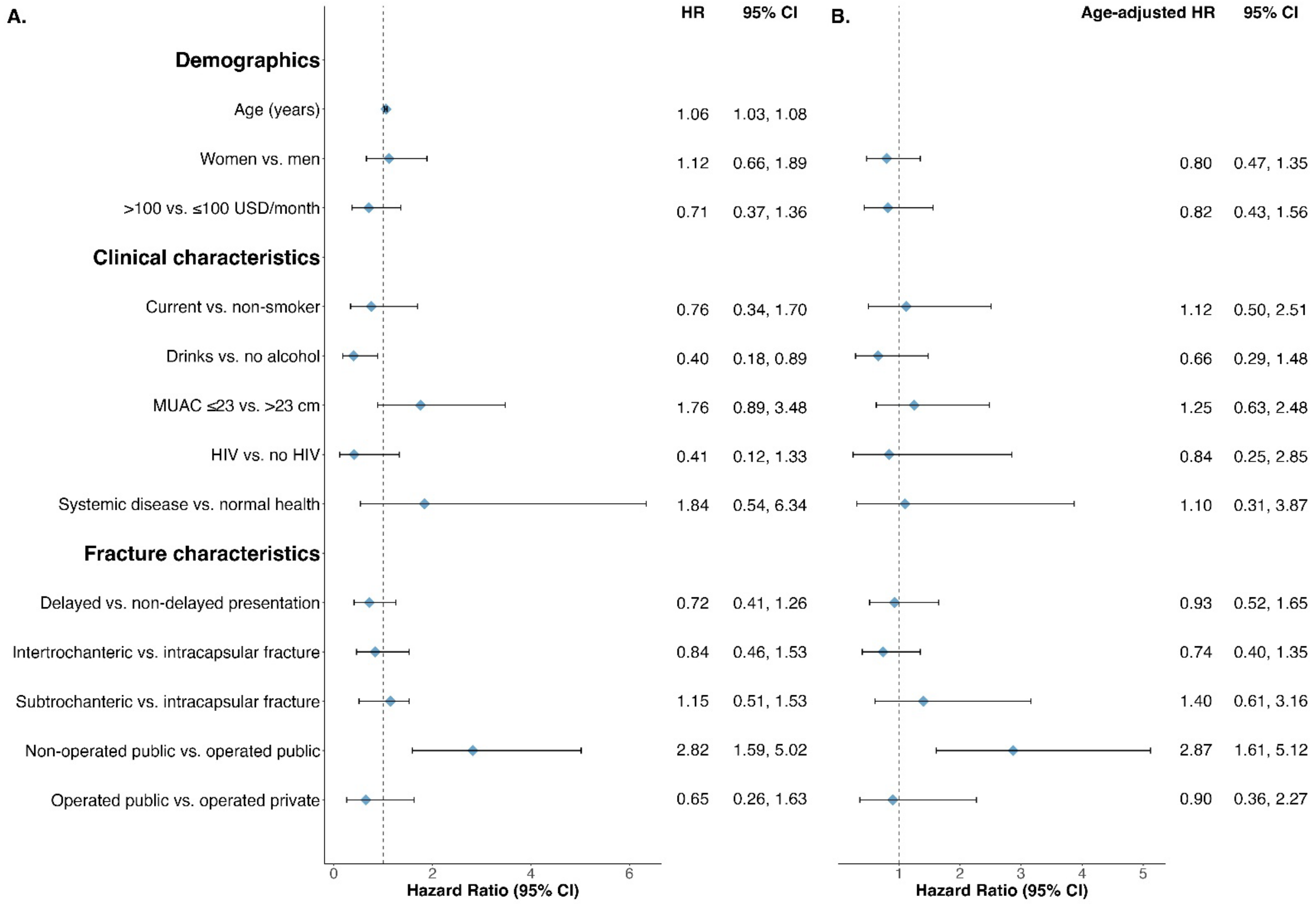

Supplementary Figure S3. Crude (panel A) and age-adjusted (panel B) hazard ratios (and 95%CI) of 1-year mortality risk following hip fracture. Mild or severe systemic disease = ASA grades II and III; normal health = ASA grade I. Note there were no patients with ASA grade IV or V. P-values and examination of log-log residual plots indicated deviation from proportional hazard assumption for delay to presentation (p = 0.006) and hip fracture classification (p = 0.031)

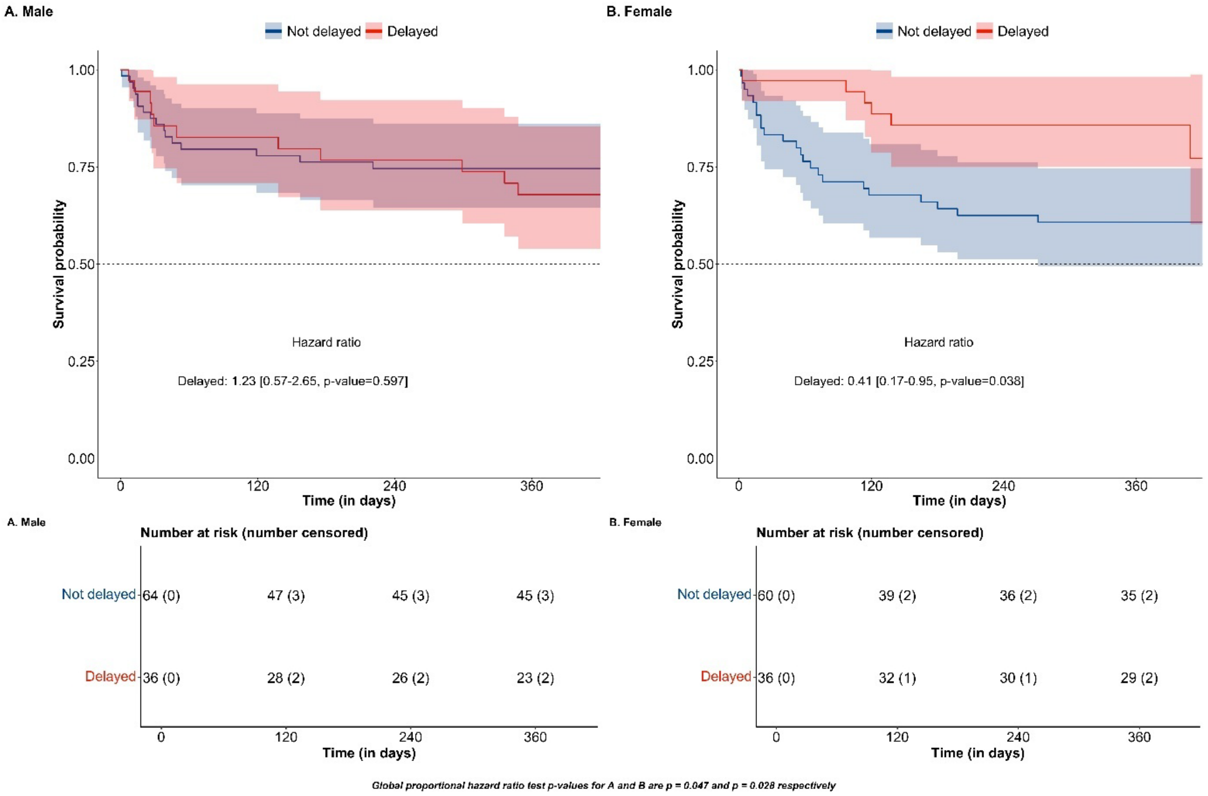

Supplementary Figure S4. Kaplan-Meier survival function estimates over 12-months for patients delayed (> 2weeks) vs not delayed (≤2weeks) for males (panel A) and females (panel B).

P-values and examination of log-log residual plots indicated some deviation from proportional hazard assumption for delay to presentation.

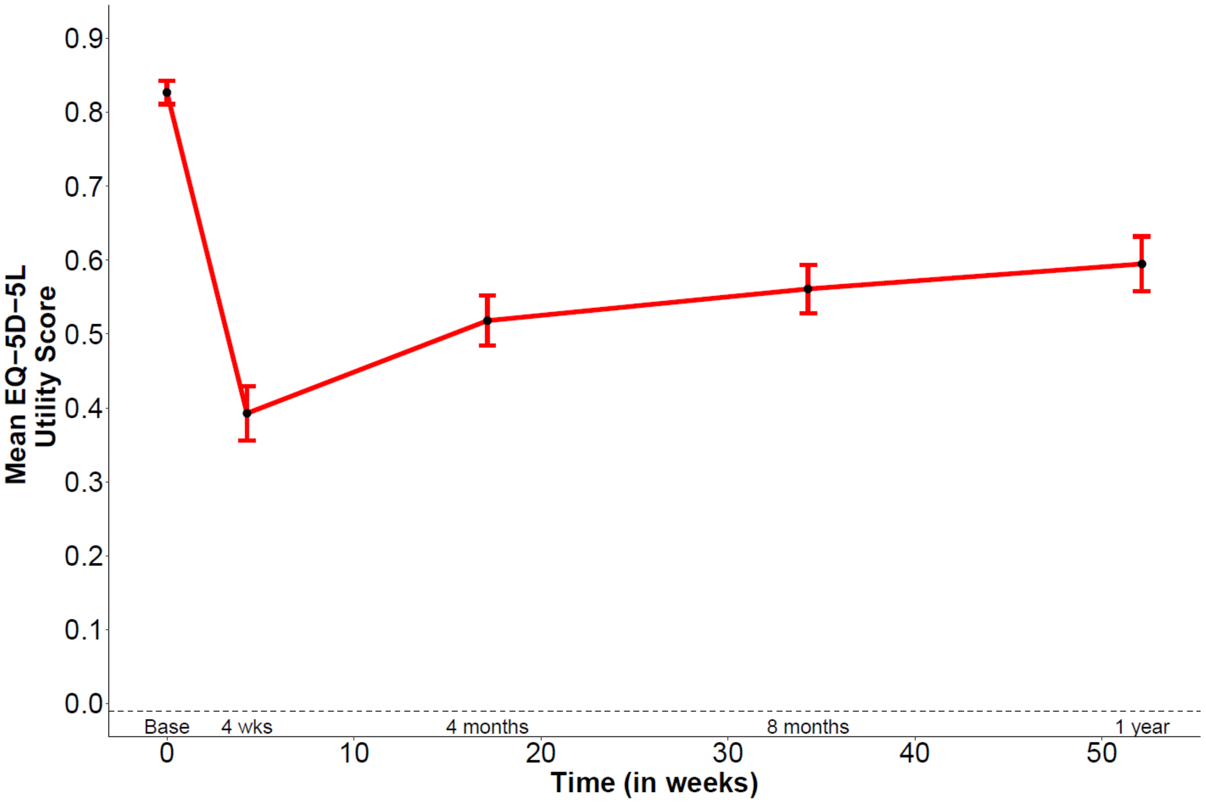

Supplementary Figure S5. Mean changes in EQ-5D-5L over 12-months for all patients, excluding 54 who died.

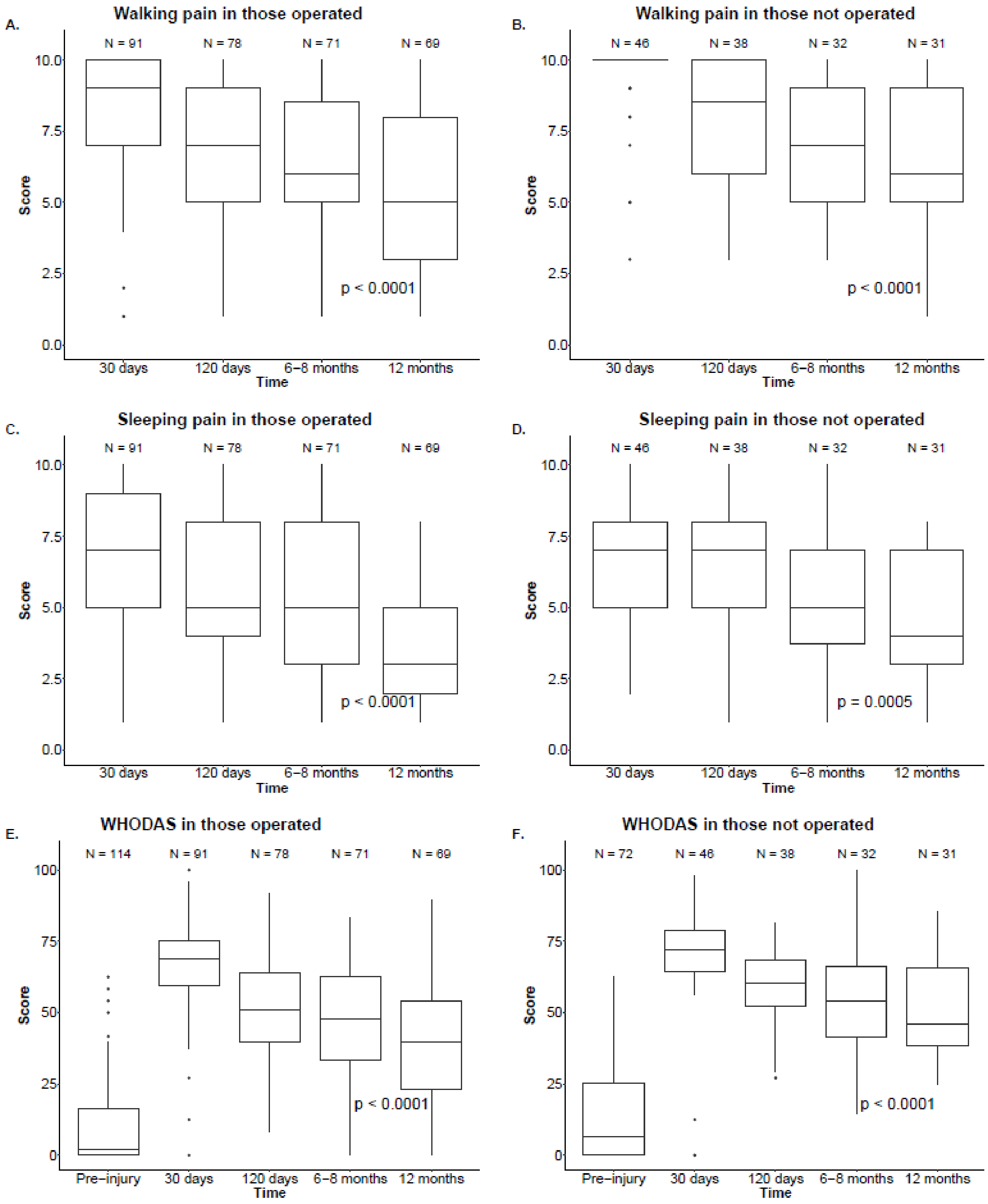

Supplementary Figure S6. Median scores for pain interfering with ability to walk in those operated (panel A) and not operated (panel B), pain interfering with ability to sleep in those operated (panel C) and not operated (panel D) and WHODAS in those operated (panel E) and not operated (panel F), at 30-days, 120-days, 6-8 months and 12-months following hip fracture. Box and Whisker plots displaying median, interquartile range and range of values.

Supplementary Table S1. Baseline characteristics of the study population stratified by consent status. Data are N(%).

	Consent not obtained	Died before consenting	Provided consent
**Hip fractures cases (N)**	36	6	190
**Patients characteristics:**			
**Age in years (median [IQR])**	75 [64,83]	73 [64,80]	74 [63,83]
**Female sex**	21 (58)	3 (50)	93 (49)
**Harare resident**	23 (64)	5 (83)	101 (53)
**Hip fracture specific characteristics:**			
**Mechanism of injury indicates low energy trauma**	32 (89)	6 (100)	156 (82)
**Presentation delayed by >2 weeks from injury**	10 (28)	1 (17)	71 (37)
**Presenting to a public hospital**	15 (42)	3 (50)	170 (89)

N=232 (all people with a hip fracture eligible for the study)

Supplementary Table S2. Baseline characteristics of the study population stratified by whether presentation delayed (>2 weeks after injury). Data are N(%), unless otherwise specified.

	Time between injury and presentation	
	**≤2 weeks**	**>2 weeks**
**Hip fractures cases**		
**Total**	124 (100)	72 (100)
**Consented**	119 (96)	71 (99)
**Died before consent**	5 (4)	1 (1)
**Patients characteristics**		
**Age in years[a] (median [IQR])**	76 (64-84)	72 (62-78.5)
**Female sex[a]**	60 (48)	36 (50)
**Harare resident[a]**	79 (64)	27 (38)
**Black African**	112 (94)	69 (97)
**Educational attainment[b]**		
**None**	12 (10)	9 (13)
**Primary**	61 (51)	34 (48)
**Secondary**	29 (24)	17 (24)
**Post-secondary**	12 (10)	5 (7)
**Marital status[b]**		
**Married or cohabiting**	51 (43)	31 (44)
**Separated/divorced/never married**	15 (13)	7 (10)
**Widowed**	52 (44)	30 (42)
**Residence type[b]**		
**Own home**	96 (81)	60 (85)
**Relative home**	17 (14)	7 (10)
**Residential/nursing home**	3 (3)	1 (1)
**Other**	3 (3)	2 (3)
**Household income[b] ≤100USD**	66 (55)	49 (69)
**Current tobacco smoker**	20 (17)	11 (15)
**Comsumes alcohol[b]**	31 (26)	20 (28)
**Living with HIV (self-reported)[b]**	13 (11)	11 (15)
**Low MUAC[b] (≤23 cm)**	22 (18)	18 (25)
**Hip fracture specific characteristics**		
**Hip fracture type[b]**		
**Intracapsular**	50 (40)	32 (44)
**Intertrochanteric**	53 (43)	26 (36)
**Subtrochanteric**	13 (10)	14 (19)
**Mechanism of injury indicates low energy trauma[a]**	104 (84)	58 (81)
**Presenting to a public hospital[a]**	105 (85)	68 (94)
**Operated[a]**	78 (63)	41 (57)
**ASA grade[b]**		
**I Normal health**	17 (14)	10 (14)
**II Mild systemic disease**	43 (36)	26 (37)
**III Severe systemic disease**	10 (8)	5 (7)
**Hospital length of stay in days (median IQR])**	20 (12-32)	24 (14-35)
**Hospital days until surgery (median [IQR]) [b]**	15 (7-26)	22 (10-31)
**Hospital days from surgery until discharge (median [IQR])[b]**	3 (2-7)	3 (2-4)

MUAC, mid-upper arm circumference; ASA, American Society of Anaesthesiologists. [a]Variables available for all 196 with a minimum dataset (those who consented and those who died before consent). Other variables available only for the consented patients N=190. [b]Missing values: educational level n=11; marital status n=4; residence type n=1; household income n=10; alcohol intake n=2; HIV n=47; MUAC n=29; hip fracture type n=8; ASA grade n=79; date of surgery n=1

Supplementary Table S3. Baseline characteristics of the study population stratified by the reporting of vital status. Data are N(%), unless otherwise specified.

	Vital status	
	**Unknown**	**Known**
**Hip fractures cases**		
**Total**	5 (100)	187 (100)
**Consented**	5 (100)	181 (97)
**Died before conset**	0 (0)	6 (3)
**Patients characteristics**		
**Age in years[a] (median [IQR])**	67 [63,72]	74 [62,83]
**Female sex[a]**	2 (40)	92 (49)
**Harare resident[a]**	1 (20)	101 (54)
**Education level[b]**		
**None**	0 (0)	21 (12)
**Primary**	3 (60)	91 (50)
**Secondary**	0 (0)	44 (24)
**Post-secondary**	1 (20)	15 (8)
**Marital status**		
**Married or cohabiting**	5 (100)	75 (41)
**Separated/divorced/never married**	0 (0)	20 (11)
**Widowed**	0 (0)	82 (45)
**Residence type**		
**Own home**	5 (100)	147 (81)
**Relative home**	0 (0)	24 (13)
**Residential/nursing home**	0 (0)	4 (2)
**Other**	0 (0)	5 (3)
**Household income[b] ≤100USD**	4 (80)	109 (60)
**Current tobacco smoker**	1 (20)	30 (17)
**Comsumes alcohol[b]**	1 (20)	48 (27)
**Living with HIV (self-reported)[b]**	0 (0)	24 (13)
**Low MUAC[b] (≤23 cm)**	1 (20)	48 (27)
**Hip fracture specific characteristics**		
**Hip fracture type[b]**		
**Intracapsular**	2 (40)	78 (42)
**Intertrochanteric**	2 (40)	76 (41)
**Subtrochanteric**	1 (20)	25 (123)
**Mechanism of injury indicates low energy trauma[a]**	4 (80)	155 (83)
**Presenting to a public hospital[a]**	5 (100)	166 (89)
**Operated[a]**	3 (60)	114 (61)
**ASA grade[b]**		
**I Normal health**	0 (0)	26 (14)
**II Mild systemic disease**	3 (60)	66 (36)
**III Severe systemic disease**	0 (0)	15 (8)
**Hospital length of stay in days (median [IQR])**	18 [14,19]	23 [13,33]
**Hospital days until surgery (median [IQR])**	12 [10,15]	18 [8,30]
**Hospital days from surgery until discharge (median [IQR])[b]**	2 (1-10)	3 (2-6)

N = 192 (All 196 with a minimum dataset minus those participants who withdrew consent for follow up (N=4)). MUAC, mid-upper arm circumference; ASA, American Society of Anaesthesiologists. [a]Variables available for all with a minimum dataset (those who consented and those who died before consent). Other variables available only for the consented patients. [b]Missing values: educational level n=11; marital status n=4; residence type n=1; household income n=9; alcohol intake n=2; HIV n=45; MUAC n=29; hip fracture type n=8; ASA grade n=76; date of surgery n=1

Supplementary Table S4. Cumulative mortality over 12-months, stratified by age (<70 vs. ≥70 years), delayed presentation (>2 weeks), and facility type and operation status.

Data are N(%)

		N	30-days	120-days	6-8 months	12-months
**All**		187	27 (14)	45 (24)	51 (27)	55 (29)
**By age**	<70 years	70	3 (4)	5 (7)	6 (9)	6 (9)
	≥70 years	117	24 (21)	40 (34)	45 (38)	49 (42)
**By presentation delay**	≤2 weeks	119	21 (18)	33 (28)	38 (32)	39 (33)
	>2 weeks	68	6 (9)	12 (18)	13 (19)	16 (24)
**By facility type and operation status**	Non-operated public	73	19 (26)	28 (38)	29 (40)	31 (42)
	Operated public	93	5 (5)	13 (14)	16 (17)	18 (19)
	Operated private	21	3 (14)	4 (19)	6 (29)	6 (29)

N = 187 (All 196 with a minimum dataset, minus 5 for whom vital status was unknown and 4 who withdrew consent)

Supplementary Table S5: Mean change in EQ-5D-5L at 30, 120days and 12-months overall, and stratified by age, delayed presentation, and by facility type and operation status

		Baseline<br>N = 184	30 days<br>N = 158	120 days<br>N = 155	12-months<br>N = 148	30 days vs. Baseline		120 days vs. Baseline		12-months vs. baseline	
		Mean<br>(95%CI)				Mean difference (95%CI)	% reduction	Mean difference (95%CI)	% reduction	Mean difference (95%CI)	% reduction
**All**		0.81<br>(0.80,0.83)	0.29<br>(0.25,0.34)	0.34<br>(0.29,0.39)	0.35<br>(0.29,0.42)	0.52<br>(0.47,0.56)	64	0.47<br>(0.42,0.52)	58	0.46<br>(0.39,0.52)	56
**By age**	<70 years	0.86<br>(0.84,0.88)	0.39<br>(0.34,0.44)	0.54<br>(0.48,0.60)	0.60<br>(0.54,0.67)	0.47<br>(0.42,0.52)	55	0.32<br>(0.26,0.38)	37	0.26<br>(0.19,0.32)	30
	≥70 years	0.78<br>(0.76,0.80)	0.24<br>(0.18,0.30)	0.22<br>(0.16,0.29)	0.19<br>(0.11,0.27)	0.54<br>(0.48,0.60)	69	0.56<br>(0.49,0.63)	71	0.59<br>(0.51,0.67)	76
**By delayed presentation**	≤2 weeks	0.80<br>(0.78,0.82)	0.29<br>(0.23,0.34)	0.31<br>(0.24,0.38)	0.34<br>(0.26,0.42)	0.52<br>(0.46,0.57)	64	0.49<br>(0.42,0.56)	61	0.46<br>(0.38,0.54)	58
	>2 weeks	0.83<br>(0.81,0.85)	0.31<br>(0.24,0.37)	0.40<br>(0.32,0.48)	0.38<br>(0.28,0.48)	0.52<br>(0.46,0.59)	63	0.43<br>(0.35,0.52)	52	0.45<br>(0.35,0.55)	54
**By facility type and operation status**	Non-operated public	0.80<br>(0.77,0.83)	0.15<br>(0.09,0.22)	0.22<br>(0.13,0.30)	0.21<br>(0.12,0.31)	0.65<br>(0.58,0.72)	81	0.58<br>(0.48,0.69)	73	0.59<br>(0.47,0.70)	74
	Operated public	0.81<br>(0.79,0.83)	0.36<br>(0.32,0.41)	0.42<br>(0.36,0.49)	0.46<br>(0.38,0.54)	0.45<br>(0.40,0.50)	55	0.39<br>(0.32,0.46)	48	0.35<br>(0.27,0.43)	43
	Operated private	0.86<br>(0.82,0.90)	0.57<br>(0.42,0.72)	0.47<br>(0.28,0.67)	0.37<br>(0.10,0.64)	0.29<br>(0.16,0.43)	34	0.39<br>(0.21,0.56)	45	0.49<br>(0.25,0.73)	57

N = 186 (All 196 with a minimum dataset, minus 6 patients who died before consent and 4 who withdrew consent for follow up). EQ5D missing for 2 at baseline

Supplementary Table S6: Pain and disability following hip fracture**.** Data are N(%)

	Pre-injury	30 days	120 days	6-8 months	12-months
**N alive**	186	165	147	141	137
**N (%) with data available**	186 (100)	137 (83)	116 (79)	103 (73)	100 (73)
**Extent of pain from hip injury**					
**All the time everyday**		23 (17)	8 (7)	4 (4)	4 (4)
**Some of the time everyday**		83 (61)	56 (48)	49 (48)	35 (35)
**Some of the time every week**		20 (15)	40 (34)	27 (26)	17 (17)
**Only now and again**		7 (5)	11 (9)	23 (22)	41 (41)
**I do not have pain anymore**		4 (3)	1 (1)	(0)	3 (3)
**Hip pain that interferes with walking in the last 24 hours**		135 (99)	114 (98)	102 (99)	94 (94)
**Hip pain that interferes with sleeping in the last 24 hours**		132 (96)	107 (92)	96 (93)	80 (80)
**Free of hip pain in the last 24 hours**		2 (1)	1 (1)	0 (0)	3 (3)
**No disability (WHODAS = 0)**	67 (36)	5 (4)	2 (2)	1 (1)	2 (2)

N = 186 (All 196 with a minimum dataset, minus 6 patients who died before consent and 4 who withdrew consent for follow up).
